# Patterns of social mixing in England changed in line with restrictions during the COVID-19 pandemic (September 2020 to April 2022)

**DOI:** 10.1038/s41598-022-14431-3

**Published:** 2022-06-21

**Authors:** Louise E. Smith, Henry W. W. Potts, Richard Amlȏt, Nicola T. Fear, Susan Michie, G. James Rubin

**Affiliations:** 1 Institute of Psychiatry, Psychology and Neuroscience, King’s College London, London, UK; 2 NIHR Health Protection Research Unit in Emergency Preparedness and Response, Weston Education Centre, King’s College London, Cutcombe Road, London, SE5 9RJ UK; 3 Institute of Health Informatics, University College London, 222 Euston Road, London, NW1 2DA UK; 4 Behavioural Science and Insights Unit, UK Health Security Agency, Porton Down, Wiltshire, Salisbury, SP4 0JG UK; 5 King’s Centre for Military Health Research and Academic Department of Military Mental Health, King’s College London, London, UK; 6 Centre for Behaviour Change, University College London, 1-19 Torrington Place, London, WC1E 7HB UK; 7 Department of Psychological Medicine, Weston Education Centre, King’s College London, Cutcombe Road, London, SE5 9RJ UK

**Keywords:** Human behaviour, Viral infection

## Abstract

Social mixing contributes to the transmission of SARS-CoV-2. We developed a composite measure for risky social mixing, investigating changes during the pandemic and factors associated with risky mixing. Forty-five waves of online cross-sectional surveys were used (n = 78,917 responses; 14 September 2020 to 13 April 2022). We investigated socio-demographic, contextual and psychological factors associated with engaging in highest risk social mixing in England at seven timepoints. Patterns of social mixing varied over time, broadly in line with changes in restrictions. Engaging in highest risk social mixing was associated with being younger, less worried about COVID-19, perceiving a lower risk of COVID-19, perceiving COVID-19 to be a less severe illness, thinking the risks of COVID-19 were being exaggerated, not agreeing that one’s personal behaviour had an impact on how COVID-19 spreads, and not agreeing that information from the UK Government about COVID-19 can be trusted. Our composite measure for risky social mixing varied in line with restrictions in place at the time of data collection, providing some validation of the measure. While messages targeting psychological factors may reduce higher risk social mixing, achieving a large change in risky social mixing in a short space of time may necessitate a reimposition of restrictions.

## Introduction

Behavioural strategies to prevent the spread of SARS-CoV-2 have focused on reducing the number of contacts made in everyday life through policies requiring people to work from home where possible, avoid hospitality and leisure venues, and remain physically distant from each other. This is an effective way of decreasing transmission^1^. In England, there have been a series of national restrictions limiting social contact (Table 1). In some cases, social mixing was only allowed in outdoor spaces, due to evidence suggesting that transmission is lower in more ventilated areas (see Supplementary Materials 1 for a more detailed description of restrictions)^2,3^. Compared to before the pandemic, people’s contacts were reduced between March 2020 and March 2021, with contact patterns changing in line with UK Government recommendations^4^. Previous studies have investigated people’s contact behaviour in a range of different settings (e.g. at home, work, on public transport, and while socialising). A previous paper from this series of surveys has explored factors associated with working outside of the home^5^. In this study, we focus solely on patterns of social mixing.

**Table 1:** Restrictions on social mixing in England during the pandemic (March 2020–July 2021).

Date	Restriction	Setting	Social contact allowed	Number of people from other households allowed	Distancing
16 March 2020	People asked to stay at home	All settings	Yes	Not specified	Not specified
23 March 2020	First national lockdown	All settings	No	None	N/A
13 May 2020	Step 1 of lockdown restrictions easing	Outdoor public spaces	Yes	One	2 m+
1 June 2020	Step 2 of lockdown restrictions easing	Outdoor public spaces	Yes	Five	2 m+
4 July 2020	Hospitality reopens	Outdoors	Yes	Five	1 m+
		Indoors	Yes	Not specified, from one other household	1 m+
14 September 2020	Rule of six	Outdoors	Yes	Five	Not specified
		Indoors	Yes	Five	Not specified
14 October 2020	**Tiers (local COVID-19 alert level)**				
	1 (medium)	Outdoors	Yes	Five	Not specified
		Indoors	Yes	Five	Not specified
	2 (high)	Outdoors	Yes	Five	Not specified
		Indoors	No	None	N/A
	3 (very high)	Outdoor public spaces	Yes	Five	Not specified
		Indoors	No	None	N/A
5 November 2020	Second national lockdown	All settings	No	None	N/A
2 December 2020	**Tiers (local COVID-19 alert level)**				
	1 (medium)	Outdoors	Yes	Five	Not specified
		Indoors	Yes	Five	Not specified
	2 (high)	Outdoors	Yes	Five	Not specified
		Indoors	No	None	N/A
	3 (very high)	Outdoor public spaces	Yes	Five	Not specified
		Indoors	No	None	N/A
19 December 2020	Tier 4	Outdoor public spaces	Yes	One	1 m+
5 January 2021	Third national lockdown	All settings	No	None	N/A
29 March 2021	Step 1 of roadmap	Outdoor public spaces	Yes	Five, or one other household	1 m+
		Indoors	No	None	N/A
12 April 2021	Step 2 of roadmap	Outdoors	Yes	Five, or one other household	1 m+
		Indoors	No	None	N/A
17 May 2021	Step 3 of roadmap	Outdoors	Yes	Twenty-nine	1 m+
		Indoors	Yes	Five, or one other household	None
19 July 2021	Step 4 of roadmap	All settings	Yes	No limit	None

Protective behaviours are only effective at preventing transmission of infection if people adhere to them. One way of encouraging uptake is to legally enforce behaviours, and to limit people’s opportunity to socialise (e.g. by closing hospitality venues). In England, legal restrictions on social mixing were in place between 27 March 2020 and 19 July 2021^6,7^. After this, emphasis was placed on individuals understanding and managing their own risk^8^. While new measures were introduced in response to the Omicron variant (November 2021 to January 2022), these did not include restrictions on social mixing, focusing instead on mandating face coverings and vaccine passports in certain indoor spaces, and working from home where possible^9,10^.

A range of factors—socio-demographic, contextual, and psychological—may affect whether people adopt protective behaviours. Research suggests that women, older people, those with chronic illnesses, people who perceived measures to be more effective, people who perceived COVID-19 to be a more severe illness, and those who thought that others were also adhering to measures were more likely to adopt physical distancing behaviours^11–14^. A study investigating close contacts during the pandemic found that people who reported more contacts were less likely to think that COVID-19 would be a serious illness for them, were more likely to agree that they were likely to catch COVID-19, and were more concerned that they might spread COVID-19 to others^4^. These studies investigated factors associated with individual dimensions of social mixing, for example the number of times people had met up with friends or family socially, whether they came into close contact with others, or self-reported adherence to Government guidelines. A detailed understanding of how patterns of social mixing changed under different restrictions over the course of the pandemic is missing. Furthermore, most studies investigated mixing at the start of the pandemic (May 2020), with one study analysing data collected up to December 2020^14^, and another analysing data up to March 2021^4^. At the time of writing, there were no available data investigating social mixing after the release of all legal restrictions in the UK on 19 July 2021.

Since the start of the COVID-19 outbreak, we have been working with the English Department of Health and Social Care to track behaviours that affect SARS-CoV-2 transmission using a series of online cross-sectional surveys. Reporting has so far focused on individual behaviours and associated factors. However, the surveys include questions asking for detailed information on participants’ latest instance of social mixing (including setting, whether they maintained distance from others, how many other households they mixed with, how many people from other households were present), all of which influence transmission risk. We used these data to:

1. develop a composite measure for risky social mixing based on most recent social mixing, taking into account setting, close contact, number of households, and number of people from other households;
2. describe change over time in the percentage of people engaging in risky social mixing;
3. identify who is most likely to engage in risky social mixing, and whether socio-demographic, psychological, and contextual factors are associated with risky social mixing.

## Methods

### Design

A series of cross-sectional surveys have been carried out by BMG Research and then Savanta (both Market Research Society Company Partners) since January 2020 on behalf of the English Department of Health and Social Care. We analysed these data as part of the COVID-19 Rapid Survey of Adherence to Interventions and Responses (CORSAIR) study^15^. For this study, we used data collected between 14 September 2020 and 13 April 2022 (waves 28 to 72). These are the waves in which all questions used to form our outcome measure were included in the survey; some had not been introduced before 14 September 2020 (wave 28).

### Participants

Participants were recruited from two specialist research panel providers, Respondi (n = 50,000) and Savanta (n = 31,500) and were eligible for the study if they were aged 16 years or over and lived in the UK (n ≈ 2000 per wave). Members of online research panels have consented to being contacted to take part in online surveys. Following industry standards, informed consent was implied by participants’ completion of the survey. Quotas based on age and gender (combined) were applied to ensure the sample was broadly representative of the UK population. After having completed the survey, participants were unable to take part in the following three waves of data collection. Participants were reimbursed in points which could be redeemed in cash, gift vouchers or charitable donations (up to 70p per survey). For this study, only participants living in England were selected, as restrictions differed between the four nations of the UK (n ≈ 1700 per wave).

### Study materials

Full survey questions are presented in Supplementary Materials 2.

#### Socio-demographic characteristics

Participants reported their age, gender, employment status, highest educational or professional qualification, ethnicity, relationship status, how many people lived in their household, their first language, whether there was a dependent child in the household, the highest earner in household worked in a manual occupation, and whether they or a household member had a chronic illness. Participants were also asked for their full postcode, from which geographical region and indices of multiple deprivation were determined^16^.

Participants were asked if they thought they had previously, or currently, had COVID-19. We recoded answers into a binary variable (“I’ve definitely had it, and had it confirmed by a test” and “I think I’ve probably had it”, vs “I don’t know whether I’ve had it or not”, “I think I’ve probably not had it”, and “I’ve definitely not had it”).

Financial hardship was measured by asking participants to what extent in the past seven days they had been struggling to make ends meet, skipping meals they would usually have, and were finding their current living situation difficult (Cronbach’s α = 0.81).

#### Risky social mixing

All participants were asked “the number of times [they had] been out of [their] home in the last seven days … to meet up with friends and/or family that [they didn’t] live with”. From 1 June 2021 (wave 51), the wording of this question was slightly changed, to ask participants “how many times [they had] done each of the following activities in the past seven days…met up with friends and/or family that [they didn’t] live with”.

Participants who indicated they had been out at least once were asked a series of follow-up questions about “the last occasion [they] met up with friends and/or family they [didn’t] live with”. Questions asked whether the last occasion participants met up with friends or family occurred indoors or outdoors (“setting”), whether they stayed at least 2 m apart (“close contact”), how many other households were present (“total number of households”), and the number of people from outside their household (“number of people from other households”). Full survey items used in our outcome measure are shown in Table 2.

**Table 2:** Coding of variables to compute risky social mixing variable.

Variable	Question text	Response options	Risk level
Setting (indoors/outdoors)	And still thinking about only the last occasion you met up with friends and/or family, were you indoors or outdoors? [People in wave 44 were asked a slightly different version of this question. Therefore, we have excluded them from analyses]	Exclusively outdoors	Lowest (“exclusively outdoors”)
		Mostly outdoors	Medium (“mostly outdoors”)
		Equally split between indoors and outdoors	Highest (“indoors”)
		Mostly indoors	Highest (“indoors”)
		Exclusively indoors	Highest (“indoors”)
Close contact	Again, thinking about the last occasion you met with friends and/or family that you don’t live with, did people stay at least 2 m apart?	Yes, at all times	Lowest (“distanced”)
		Yes, most of the time	Lowest (“distanced”)
		Yes, some of the time	Highest (“not distanced”)
		No—not at all	Highest (“not distanced”)
Total number of households	The last time you met with friends and/ or family that you don’t live with, how many households (not people) did those people come from? Don’t include your own household in this number	Scale	Question + 1 (to include own household)
			Lowest (“2”)
			Highest (“3+”)
Number of people from other households	And still thinking about the last time you met friends and/or family that you don’t live with, how many people from outside your household were there?	Scale	Lowest (“ ≤ 2”)
			Highest (“ ≥ 3”)

#### Contextual and psychological factors

Participants were asked “the number of times [they had] been out of [their] home in the last seven days … to go out to work”. On 1 June 2021 (wave 51), the wording of this question was slightly changed, to ask participants “how many times [they had] done each of the following activities in the past seven days … left the house to go out to work (number of days)”.

Worry about COVID-19 was measured by asking participants “overall, how worried [they were] about coronavirus” on a five-point scale from “extremely worried” to “not at all worried”. Perceived risk of COVID-19 was measured by asking participants “to what extent [they thought] coronavirus [posed] a risk to” people in the UK and themselves personally on a five-point scale from “major risk” to “no risk at all”.

Other psychological factors were measured using a series of five statements, each of which was measured on a five-point scale from “strongly agree” to “strongly disagree”. Statements asked participants to what extent they agreed that COVID-19 would be a serious illness for them, they would worry about what others would think of them if they tested positive for COVID-19, someone could spread COVID-19 to other people even if they did not have symptoms yet, their personal behaviour had an impact on how COVID-19 spread, and they thought the risks of COVID-19 were being exaggerated.

Participants also indicated the extent to which they thought information from the UK Government about COVID-19 (a) could be trusted, and (b) was biased or one-sided on a five-point scale from “strongly agree” to “strongly disagree”.

### Ethics

This work was conducted as a service evaluation of the Department of Health and Social Care’s public communications campaign and, following advice from King’s College London Research Ethics Committee, was exempt from requiring ethical approval. The study was otherwise carried out in accordance with the Declaration of Helsinki.

### Power

A sample size of 1700 allows a 95% confidence interval of about plus or minus 2% for the prevalence estimate for a survey item with a prevalence of 50%.

### Analysis

**Figure 1:**
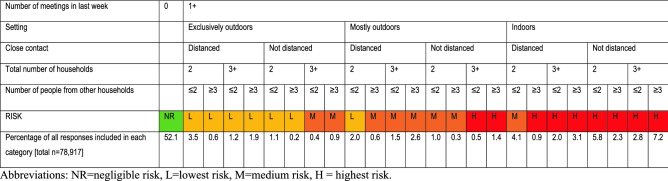
Categorisation of risk ratings.

We used information about participants’ latest instance of social mixing to compute a measure of “risky social mixing”. Participants who reported that they had not been out to meet friends or family that they did not live with were assigned to the “negligible risk” category. For participants who reported having been out to meet friends or family from another household, we used information about setting (indoors/outdoors), whether they came into close contact with others, the total number of households, and how many people from other households were present to assign them a risk category. First, we created dichotomous or trichotomous variables denoting low or high risk (or low, medium, high risk where trichotomous) for each factor (Table 2). Second, participants were categorised according to risk ratings for individual factors (setting, close contact, total number of households present, number of people from other households). Third, risk ratings for individual variables were combined to give an overall risk rating, and participants’ latest instance of social mixing was categorised as “lowest risk”, “medium risk” or “highest risk” (Fig. 1). Risk ratings and their categorisations were developed using consensus agreement between the authors and the English Department of Health and Social Care and with advice from participants in COVID-19 working groups from public health agencies and experts in infectious disease transmission and modelling. People in wave 44 (data collected 22 to 23 February 2021) were asked a slightly different version of one of the questions used to create our composite measure. Therefore, we have excluded them from analyses. See Supplementary Materials 3 for responses to individual items.

To describe change in risky social mixing over time, we computed risky social mixing for each survey wave, presenting results graphically. Rates of risky social mixing between 1 November and 16 December 2021 (5 waves, wave 61 to 64) have been reported elsewhere^17^.

We investigated associations with highest risk social mixing at different time points within the pandemic. We selected slices of data (two or three survey waves) that were collected at seven different time points in the pandemic, choosing times when different restrictions were in place. Time points were: (1) rule of six indoors and outdoors (data collected 14 to 30 September 2020), (2) second national lockdown (data collected 9 to 25 November 2020), (3) third national lockdown (data collected 11 January to 9 February 2021), (4) rule of six outdoors, no indoor mixing (data collected 19 April to 5 May 2021), (5) rule of six indoors, up to 30 people outdoors (data collected 1 to 15 June 2021), (6) straight after the legal restrictions on social mixing were lifted (data collected 26 July to 10 August 2021), and (7) after the legal restriction to self-isolate if symptomatic had been lifted (proxy for return to most “normal” context in UK since the start of the pandemic; data collected 14 March to 13 April 2022).

We investigated associations between highest risk social mixing and:

1. socio-demographic characteristics (survey wave, region, gender, age [raw and quadratic], presence of a dependent child in the household, having a chronic illness oneself, having a household member who has chronic illness, employment status, highest earner in household works in a manual occupation, index of multiple deprivation, highest educational or professional qualification, ethnicity, first language, living alone, relationship status, having had COVID-19 before, and financial hardship) and,
2. contextual and psychological characteristics (having been out to work in the last week, worry about COVID-19, perceived risk of COVID-19 [to self and people in the UK], perceived severity of COVID-19, worry about what others would think if you tested positive for COVID-19, agreeing that someone can spread COVID-19 even if asymptomatic, thinking that your personal behaviour as an impact on the spread of COVID-19, thinking that the risks of COVID-19 are exaggerated, agreeing that information from the UK Government about COVID-19 can be trusted, and is biased or one-sided).

We used multivariable logistic regressions adjusting for all socio-demographic characteristics.

Multiple analyses were run on individual outcomes (n = 12), therefore we applied a Bonferroni correction (*p* ≤ 0.004).

## Results

### Participants

Descriptions of risky social mixing are based on 78,917 responses. 53.1% of respondents were women (n = 41,923; men 46.5%, n = 36,728; prefer to self-describe 0.2%, n = 196; prefer not to say 0.1%, n = 70). Respondents’ mean age was 48.4 years (SD = 17.7, range 16 to over 100 years). Respondents were slightly more likely to be white than the general population (82.5% white British, n = 65,122; 5.9% white other, n = 4638; 2.6% mixed, n = 2021; 5.3% Asian/Asian British, n = 4210; 2.6% Black/Black British, n = 2018; 0.5% Arab/other, n = 432; 0.6% prefer not to say, n = 476 [compared to 86.0% white in the 2011 census of England and Wales^18^]). Responses within each of our time slices are from separate individuals. However, respondents may appear in more than one time slice (n = 19,201 participants appear in one time point, 65.5% of responses; n = 4186 participants appear in more than one time point, 34.5% of responses).

Participants in different time slices did not vary significantly by key socio-demographic characteristics (Table 3). Where differences were statistically significant, values only differed minimally (by 4.1% or less). An exception was the increasing percentage of people who reported that they thought they had had COVID-19 in later time slices. This is congruent with the second wave of infections over winter 2020/2021. The other exception was for workplace attendance, which was markedly lower in the third national lockdown and higher after the legal obligation to self-isolate had been removed.

**Table 3:** Respondent socio-demographic characteristics and workplace attendance for the full study sample, and within each time slice.

		Full study sample (total n = 78,917), % (n)	Rule of six indoors and outdoors (total n = 3423), % (n)	Second national lockdown (total n = 5223), % (n)	Third national lockdown (total n = 5116), % (n)	Rule of six outdoors, no indoor mixing (total n = 3298), % (n)	Rule of six indoors, up to 30 people outdoors (total n = 3367), % (n)	No restrictions on social mixing (total n = 3438), % (n)	No legal obligation to self-isolate (total n = 5455), % (n)	*p*-value between time slices
Region	East Midlands	9.0 (7124)	8.4 (289)	9.4 (491)	9.1 (468)	9.3 (308)	9.1 (307)	8.8 (303)	9.2 (504)	0.90
	East of England	11.4 (9011)	10.5 (358)	11.7 (610)	12.0 (613)	12.2 (403)	11.4 (383)	10.8 (372)	11.2 (611)	
	London	14.1 (11,116)	14.6 (501)	14.1 (736)	14.0 (714)	13.3 (440)	14.2 (478)	14.2 (489)	14.0 (761)	
	North East	5.2 (4068)	5.1 (173)	5.8 (302)	5.2 (266)	5.2 (172)	5.3 (177)	5.1 (175)	5.3 (289)	
	North West	13.3 (10,510)	13.6 (466)	13.1 (683)	12.9 (658)	13.2 (436)	12.6 (425)	14.2 (488)	13.6 (742)	
	South East	15.9 (12,512)	15.1 (517)	15.4 (802)	16.0 (819)	15.1 (497)	15.9 (535)	15.1 (518)	15.7 (857)	
	South West	10.2 (8081)	10.2 (349)	10.0 (522)	10.4 (531)	9.9 (328)	10.4 (350)	10.8 (371)	9.8 (534)	
	West Midlands	10.6 (8360)	11.2 (382)	10.3 (539)	9.8 (499)	10.8 (357)	10.3 (348)	10.5 (362)	11.1 (603)	
	Yorkshire and The Humber	10.3 (8135)	11.3 (388)	10.3 (538)	10.7 (548)	10.8 (357)	10.8 (364)	10.5 (360)	10.2 (554)	
Gender	Male	46.7 (36,728)	45.8 (1563)	46.7 (2434)	47.4 (2419)	46.8 (1538)	45.8 (1536)	46.8 (1605)	48.2 (2615)	0.28
	Female	53.3 (41,923)	54.2 (1852)	53.3 (2774)	52.6 (2682)	53.2 (1751)	54.2 (1816)	53.2 (1828)	51.8 (2815)	
Age	Mean, SD	M = 48.4, SD = 17.7	M = 48.0, SD = 16.9	M = 48.6, SD = 17.1	M = 48.8, SD = 17.5	M = 47.6, SD = 16.9	M = 48.5, SD = 17.3	M = 49.4, SD = 17.3	M = 48.6, SD = 17.1	< 0.001*
	16 to 24 years	10.7 (8463)	8.9 (306)	9.5 (498)	9.9 (505)	8.9 (295)	9.6 (324)	9.2 (317)	13.4 (731)	
	25 to 34 years	16.1 (12,686)	17.0 (581)	15.1 (788)	15.5 (794)	17.6 (579)	15.9 (536)	14.8 (509)	17.8 (971)	
	35 to 44 years	16.3 (12,871)	18.5 (633)	17.7 (925)	16.3 (836)	18.3 (604)	16.9 (570)	16.4 (564)	15.2 (829)	
	45 to 54 years	18.1 (14,281)	19.3 (662)	18.6 (971)	18.7 (955)	19.7 (651)	18.1 (608)	18.9 (651)	16.9 (920)	
	55 to 64 years	16.4 (12,912)	15.0 (514)	17.3 (901)	17.1 (876)	16.6 (548)	17.8 (598)	17.6 (605)	13.7 (745)	
	65 to 74 years	14.6 (11,498)	14.8 (506)	15.6 (815)	15.0 (768)	12.6 (414)	15.4 (519)	14.4 (494)	12.9 (706)	
	75+ years	7.9 (6206)	6.5 (221)	6.2 (325)	7.5 (382)	6.3 (207)	6.3 (212)	8.7 (298)	10.1 (553)	
Dependent child in household	None	67.5 (53,308)	65.8 (2253)	68.4 (3574)	67.9 (3473)	65.6 (2164)	66.9 (2254)	67.8 (2332)	64.5 (3519)	< 0.001*
	Child present	32.5 (25,609)	34.2 (1170)	31.6 (1649)	32.1 (1643)	34.4 (1134)	33.1 (1113)	32.2 (1106)	35.5 (1936)	
Chronic illness (self)	No	71.2 (53,738)	70.1 (2356)	71.1 (3628)	72.3 (3626)	72.1 (2310)	71.6 (2359)	70.7 (2369)	70.5 (3769)	0.22
	Yes	28.8 (21,730)	29.9 (1003)	28.9 (1474)	27.7 (1391)	27.9 (892)	28.4 (935)	29.3 (984)	29.5 (1579)	
Household member has chronic illness	No	84.6 (63,864)	83.9 (2817)	83.7 (4268)	83.7 (4199)	84.6 (2708)	84.6 (2787)	84.7 (2839)	85.8 (4587)	0.05
	Yes	15.4 (11,604)	16.1 (542)	16.3 (834)	16.3 (818)	15.4 (494)	15.4 (507)	15.3 (514)	14.2 (761)	
Employment status	Not working	44.8 (34,865)	44.4 (1500)	45.0 (2314)	44.5 (2248)	42.5 (1379)	45.1 (1499)	44.7 (1518)	43.0 (2317)	0.12
	Working	55.2 (43,001)	55.6 (1880)	55.0 (2830)	55.5 (2807)	57.5 (1867)	54.9 (1824)	55.3 (1876)	57.0 (3077)	
Highest earner in household works in a manual occupation	No	71.6 (55,239)	71.3 (2386)	70.7 (3618)	71.9 (3599)	70.6 (2279)	72.3 (2383)	70.8 (2377)	73.1 (3906)	0.06
	Yes	28.4 (21,873)	28.7 (960)	29.3 (1497)	28.1 (1408)	29.4 (951)	27.7 (912)	29.2 (982)	26.9 (1436)	
Index of multiple deprivation	1st (least) to 4th quartile (most deprived), mean, SD	M = 2.66, SD = 1.1	M = 2.61, SD = 1.1	M = 2.62, SD = 1.1	M = 2.56, SD = 1.1	M = 2.64, SD = 1.09	M = 2.61, SD = 1.1	M = 2.61, SD = 1.11	M = 2.62, SD = 1.1	< 0.001*
Highest educational or professional qualification	Less than degree	67.0 (52,856)	67.5 (2311)	65.4 (3417)	65.6 (3357)	66.5 (2192)	67.1 (2259)	66.7 (2292)	67.2 (3667)	0.24
	Degree or higher	33.0 (26,061)	32.5 (1112)	34.6 (1806)	34.4 (1759)	33.5 (1106)	32.9 (1108)	33.3 (1146)	32.8 (1788)	
Ethnicity	White British	83.0 (65,122)	84.7 (2883)	83.5 (4330)	84.1 (4284)	81.2 (2658)	81.8 (2742)	84.6 (2890)	81.1 (4397)	< 0.001*
	White other	5.9 (4638)	6.5 (221)	6.9 (360)	6.7 (339)	7.4 (242)	6.0 (202)	5.5 (189)	5.1 (275)	
	Black and minority ethnicity	11.1 (8681)	8.8 (299)	9.6 (498)	9.2 (471)	11.4 (374)	12.1 (407)	9.9 (337)	13.8 (748)	
First language	Not English	8.5 (6713)	8.0 (274)	8.7 (457)	8.5 (437)	9.5 (314)	8.8 (295)	7.2 (248)	9.1 (494)	0.02
	English	91.5 (72,204)	92.0 (3149)	91.3 (4766)	91.5 (4679)	90.5 (2984)	91.2 (3072)	92.8 (3190)	90.9 (4961)	
Living alone	Not living alone	79.6 (62,808)	81.1 (2775)	80.2 (4190)	80.4 (4113)	80.3 (2648)	79.4 (2672)	77.0 (2647)	79.3 (4328)	0.001*
	Living alone	20.4 (16,109)	18.9 (648)	19.8 (1033)	19.6 (1003)	19.7 (650)	20.6 (695)	23.0 (791)	20.7 (1127)	
Relationship status	Not partnered	40.4 (31,599)	39.2 (1323)	38.7 (1995)	38.8 (1969)	39.3 (1283)	40.0 (1332)	40.4 (1372)	42.0 (2279)	0.009
	Partnered	59.6 (46,607)	60.8 (2053)	61.3 (3161)	61.2 (3104)	60.7 (1984)	60.0 (1996)	59.6 (2028)	58.0 (3146)	
Ever had COVID-19	Think not	78.2 (61,694)	84.6 (2895)	85.5 (4464)	83.6 (4278)	82.3 (2714)	82.8 (2788)	79.8 (2745)	58.1 (3172)	< 0.001*
	Think yes	21.8 (17,223)	15.4 (528)	14.5 (759)	16.4 (838)	17.7 (584)	17.2 (579)	20.2 (693)	41.9 (2283)	
Financial hardship	Range 3 (least) to 15 (most), mean, SD	N = 76,811, M = 7.7, SD = 3.1	N = 3312, M = 7.9, SD = 3.0	N = 5009, M = 7.9, SD = 3.0	N = 4898, M = 7.9, SD = 2.9	N = 3239, M = 7.6, SD = 3.1	N = 3311, M = 7.5, SD = 3.1	N = 3376, M = 7.4, SD = 3.2	N = 5009, M = 7.9, SD = 3.0	< 0.001*
Been out to work in last week	No	63.3 (49,940)	64.9 (2221)	68.5 (3576)	73.7 (3772)	62.6 (2065)	61.4 (2066)	60.4 (2077)	54.5 (2975)	< 0.001*
	Yes	36.7 (28,987)	35.1 (1202)	31.5 (1647)	26.3 (1344)	37.4 (1233)	38.6 (1301)	39.6 (1361)	45.5 (2480)	

*p ≤ 0.004.

### Social mixing

**Figure 2:**
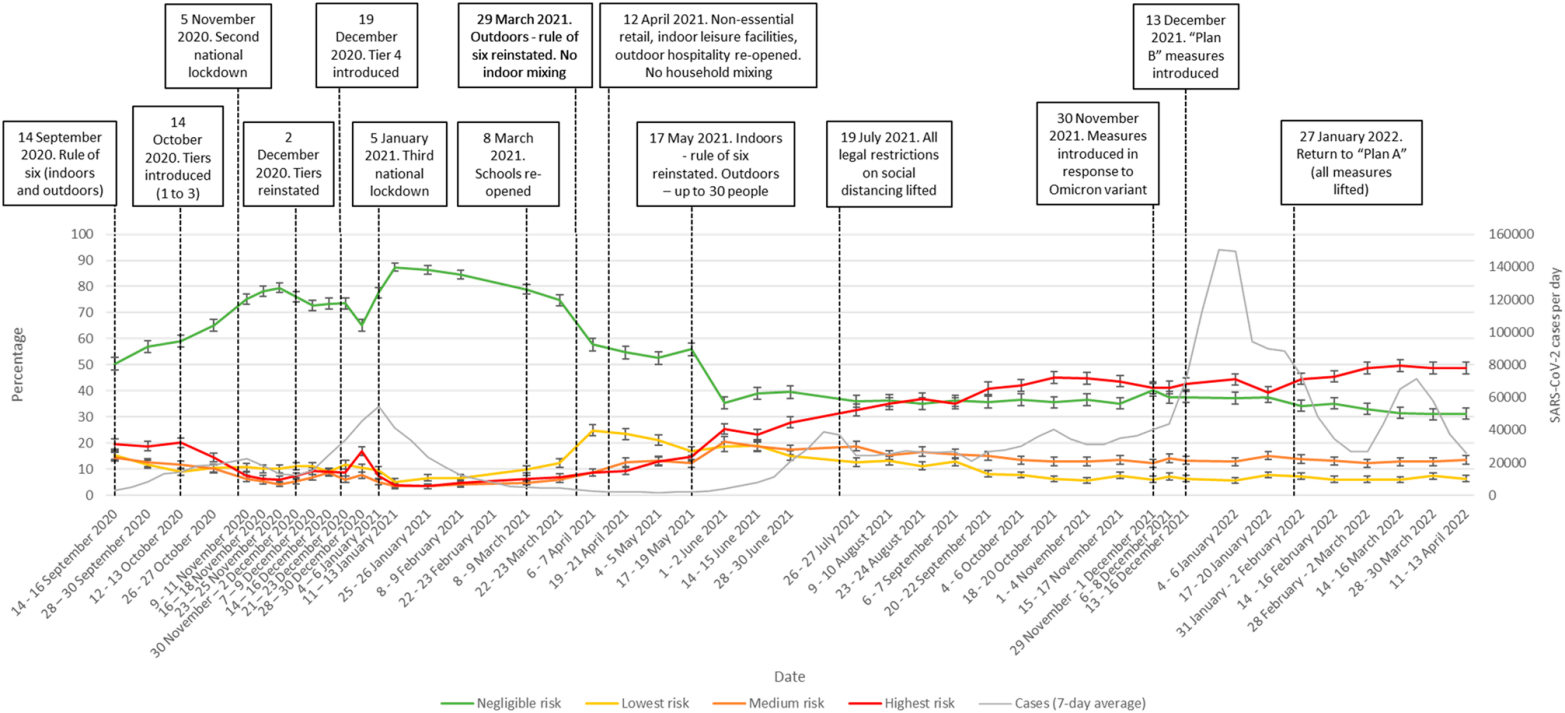
Pattern of risky social mixing between September 2020 and April 2022. Error bars are 95% confidence intervals. The grey line shows the number of new SARS-CoV-2 cases per day (7-day average) in England^19^. Case rates from April 2022 are an underestimate as only selected people were eligible for testing.

Patterns of risky social mixing changed over time, largely in line with restrictions on social mixing in place at the time of data collection (Fig. 2).

There was a large influence of time on engagement in highest risk social mixing (Table 4).

**Table 4:** Percentage of people engaging in risky social mixing at different time points in the pandemic.

	Rule of six indoors and outdoors (total n = 3423), % (n)		Second national lockdown (total n = 5223), % (n)		Third national lockdown (total n = 5116), % (n)		Rule of six outdoors, no indoor mixing (total n = 3298), % (n)		Rule of six indoors, up to 30 people outdoors (total n = 3367), % (n)		No restrictions on social mixing (total n = 3438), % (n)		No legal obligation to self-isolate (total n = 5455), % (n)		*p*-value
	% (95% CI)	n	% (95% CI)	n	% (95% CI)	n	% (95% CI)	n	% (95% CI)	n	% (95% CI)	n	% (95% CI)	n	
Negligible risk	53.6 (51.9 to 55.3)	1835	77.6 (76.5 to 78.8)	4055	86.1 (85.2 to 87.1)	4407	53.6 (51.9 to 55.3)	1769	37.2 (35.6 to 38.9)	1254	36.2 (34.6 to 37.8)	1244	31.3 (30.0 to 32.5)	1706	< 0.001
Lowest risk	13.6 (12.4 to 14.7)	465	10.3 (9.5 to 11.1)	538	6.2 (5.6 to 6.9)	318	22.3 (20.9 to 23.8)	737	18.8 (17.5 to 20.1)	632	12.9 (11.8 to 14.0)	443	6.6 (5.9 to 7.3)	360	< 0.001
Medium risk	13.6 (12.4 to 14.7)	464	5.3 (4.7 to 6.0)	279	3.6 (3.1 to 4.1)	186	13.0 (11.8 to 14.1)	428	19.6 (18.3 to 20.9)	660	17.1 (15.8 to 18.3)	587	13.1 (12.2 to 14.0)	714	< 0.001
Highest risk	19.3 (17.9 to 20.6)	659	6.7 (6 to 7.4)	351	4.0 (3.5 to 4.5)	205	11.0 (10.0 to 12.1)	364	24.4 (22.9 to 25.8)	821	33.9 (32.3 to 35.4)	1164	49.0 (47.7 to 50.4)	2675	< 0.001

At all timepoints, engaging in highest risk social mixing was associated with being less worried about COVID-19, perceiving a smaller risk of COVID-19 to oneself and people in the UK, lower perceived severity of COVID-19 to oneself, and thinking the risks of COVID-19 were being exaggerated (Tables 5, 6).

**Table 5:** Socio-demographic characteristics associated with engaging in highest risk social mixing at different time points in the pandemic.

Attribute	Level	Rule of six indoors and outdoors		Second national lockdown		Third national lockdown		Rule of six outdoors, no indoor mixing		Rule of six indoors, up to 30 people outdoors		No legal restrictions		No legal obligation to self-isolate	
		aOR for engaging in highest risk social mixing (95% CI)^†^	*p*	aOR for engaging in highest risk social mixing (95% CI)^†^	*p*	aOR for engaging in highest risk social mixing (95% CI)^†^	*p*	aOR for engaging in highest risk social mixing (95% CI)^†^	*p*	aOR for engaging in highest risk social mixing (95% CI)^†^	*p*	aOR for engaging in highest risk social mixing (95% CI)^†^	*p*	aOR for engaging in highest risk social mixing (95% CI)^†^	*p*
Survey wave in timepoint	Wave 1	Ref	–	Ref	–	Ref	–	Ref	–	Ref	–	Ref	–	Ref	–
	Wave 2	0.93 (0.77 to 1.11)	0.40	0.85 (0.64 to 1.12)	0.25	0.82 (0.55 to 1.21)	0.32	1.57 (1.24 to 1.99)	< 0.001*	0.87 (0.73 to 1.02)	0.09	1.14 (0.98 to 1.32)	0.09	0.96 (0.83 to 1.10)	0.52
	Wave 3	–	–	0.81 (0.61 to 1.08)	0.16	1.25 (0.88 to 1.79)	0.22	–	–	–	–	–	–	0.97 (0.85 to 1.12)	0.71
	Overall	–	–	χ^2^(2) = 2.3	0.32	χ^2^(2) = 5.0	0.08	–	–	–	–	–	–	χ^2^(2) = 0.4	0.81
Region	East Midlands	Ref	–	Ref	–	Ref	–	Ref	–	Ref	–	Ref	–	Ref	–
	East of England	0.93 (0.62 to 1.40)	0.74	1.50 (0.91 to 2.47)	0.11	0.98 (0.48 to 2.02)	0.96	1.20 (0.71 to 2.02)	0.50	1.09 (0.76 to 1.57)	0.64	1.26 (0.90 to 1.77)	0.17	1.13 (0.88 to 1.44)	0.35
	London	0.90 (0.61 to 1.34)	0.61	1.02 (0.62 to 1.70)	0.93	1.07 (0.55 to 2.07)	0.84	1.11 (0.66 to 1.85)	0.70	0.99 (0.69 to 1.44)	0.97	1.14 (0.82 to 1.58)	0.44	1.07 (0.84 to 1.37)	0.56
	North East	0.91 (0.55 to 1.49)	0.70	0.79 (0.39 to 1.60)	0.52	1.11 (0.47 to 2.63)	0.81	1.28 (0.68 to 2.40)	0.45	1.64 (1.07 to 2.52)	0.02	1.14 (0.75 to 1.72)	0.54	1.49 (1.09 to 2.02)	0.01
	North West	0.59 (0.39 to 0.89)	0.01	0.85 (0.49 to 1.45)	0.54	1.34 (0.69 to 2.61)	0.39	0.80 (0.47 to 1.38)	0.43	0.89 (0.62 to 1.29)	0.55	0.94 (0.68 to 1.30)	0.72	1.17 (0.92 to 1.49)	0.19
	South East	0.87 (0.60 to 1.28)	0.49	1.14 (0.69 to 1.88)	0.60	1.00 (0.51 to 1.97)	1.00	1.62 (1.00 to 2.64)	0.05	0.89 (0.63 to 1.26)	0.51	1.13 (0.82 to 1.55)	0.46	1.16 (0.92 to 1.46)	0.22
	South West	1.22 (0.82 to 1.82)	0.32	1.03 (0.59 to 1.79)	0.92	0.65 (0.28 to 1.47)	0.30	1.26 (0.74 to 2.16)	0.40	1.06 (0.73 to 1.54)	0.74	0.98 (0.70 to 1.38)	0.92	1.02 (0.79 to 1.32)	0.86
	West Midlands	0.89 (0.60 to 1.33)	0.57	1.29 (0.77 to 2.16)	0.33	1.38 (0.70 to 2.74)	0.35	1.03 (0.60 to 1.77)	0.91	1.22 (0.84 to 1.76)	0.30	0.92 (0.66 to 1.30)	0.65	1.18 (0.92 to 1.51)	0.20
	Yorkshire and The Humber	0.94 (0.63 to 1.40)	0.76	1.15 (0.68 to 1.95)	0.60	1.43 (0.73 to 2.79)	0.30	1.28 (0.76 to 2.17)	0.36	1.18 (0.82 to 1.71)	0.37	1.13 (0.80 to 1.59)	0.49	1.17 (0.91 to 1.52)	0.22
	Overall	χ^2^(8) = 14.1	0.08	χ^2^(8) = 8.6	0.38	χ^2^(8) = 6.6	0.58	χ^2^(8) = 10.8	0.21	χ^2^(8) = 13.7	0.09	χ^2^(8) = 6.9	0.54	χ^2^(8) = 8.6	0.38
Gender	Male	Ref	–	Ref	–	Ref	–	Ref	–	Ref	–	Ref	–	Ref	–
	Female	1.09 (0.90 to 1.31)	0.38	0.80 (0.63 to 1.01)	0.06	0.58 (0.42 to 0.79)	0.001*	0.85 (0.67 to 1.07)	0.17	1.11 (0.93 to 1.31)	0.24	1.17 (1.00 to 1.36)	0.05	1.44 (1.29 to 1.61)	< 0.001*
Age (per decade)	Raw age	0.89 (0.83 to 0.95)	0.001*	0.88 (0.80 to 0.96)	0.004*	0.84 (0.75 to 0.94)	0.003*	0.86 (0.79 to 0.94)	0.001*	0.92 (0.86 to 0.98)	0.01	0.93 (0.87 to 0.99)	0.01	0.92 (0.88 to 0.96)	< 0.001*
Age: quadratic (age–mean)^2^	–	1.0004 (1.0000 to 1.0007)	0.03	1.0004 (0.9999 to 1.0008)	0.09	1.0005 (0.9999 to 1.0010)	0.09	1.0008 (1.0003 to 1.0012)	< 0.001*	1.0002 (0.9999 to 1.0005)	0.29	1.0001 (0.9998 to 1.0003)	0.63	1.0001 (1.0000 to 1.0003)	0.13
Dependent child in household	None	Ref	–	Ref	–	Ref	–	Ref	–	Ref	–	Ref	–	Ref	–
	Child present	1.08 (0.87 to 1.35)	0.49	1.16 (0.87 to 1.55)	0.30	1.21 (0.83 to 1.77)	0.31	1.16 (0.88 to 1.55)	0.30	1.02 (0.83 to 1.26)	0.85	0.94 (0.77 to 1.13)	0.50	0.97 (0.84 to 1.12)	0.66
Chronic illness (self)	No	Ref	–	Ref	–	Ref	–	Ref	–	Ref	–	Ref	–	Ref	–
	Yes	0.8 (0.65 to 1.00)	0.05	0.78 (0.58 to 1.04)	0.09	1.33 (0.94 to 1.89)	0.11	0.88 (0.66 to 1.16)	0.37	1.04 (0.86 to 1.26)	0.70	0.84 (0.70 to 1.00)	0.05	0.76 (0.67 to 0.87)	< 0.001*
Household member has chronic illness	No	Ref	–	Ref	–	Ref	–	Ref	–	Ref	–	Ref	–	Ref	–
	Yes	0.86 (0.66 to 1.12)	0.27	1.12 (0.81 to 1.56)	0.50	0.96 (0.61 to 1.53)	0.87	0.81 (0.56 to 1.16)	0.24	0.98 (0.77 to 1.25)	0.89	0.88 (0.70 to 1.09)	0.23	1.11 (0.94 to 1.31)	0.20
Employment status	Not working	Ref	–	Ref	–	Ref	–	Ref	–	Ref	–	Ref	–	Ref	–
	Working	0.96 (0.77 to 1.19)	0.70	0.76 (0.58 to 1.00)	0.05	1.21 (0.83 to 1.76)	0.32	0.90 (0.68 to 1.19)	0.45	0.90 (0.74 to 1.10)	0.31	0.81 (0.67 to 0.97)	0.03	0.84 (0.73 to 0.97)	0.02
Highest earner in household works in a manual occupation	No	Ref	–	Ref	–	Ref	–	Ref	–	Ref	–	Ref	–	Ref	–
	Yes	1.16 (0.94 to 1.43)	0.16	1.02 (0.78 to 1.33)	0.90	1.17 (0.83 to 1.65)	0.38	1.22 (0.94 to 1.58)	0.14	0.92 (0.75 to 1.12)	0.39	1.03 (0.87 to 1.23)	0.71	0.95 (0.83 to 1.09)	0.48
Index of multiple deprivation	1st (least) to 4th quartile (most deprived)	1.04 (0.95 to 1.13)	0.43	1.06 (0.95 to 1.19)	0.30	1.07 (0.92 to 1.24)	0.37	1.14 (1.02 to 1.29)	0.02	0.95 (0.88 to 1.03)	0.20	0.93 (0.87 to 1.01)	0.07	1.11 (1.05 to 1.18)	< 0.001*
Highest educational or professional qualification	Less than degree	Ref	–	Ref	–	Ref	–	Ref	–	Ref	–	Ref	–	Ref	–
	Degree or higher	0.98 (0.79 to 1.20)	0.83	0.81 (0.62 to 1.05)	0.11	1.10 (0.79 to 1.54)	0.57	0.95 (0.73 to 1.24)	0.70	0.99 (0.82 to 1.20)	0.92	0.86 (0.73 to 1.02)	0.09	0.95 (0.84 to 1.08)	0.43
Ethnicity	White British	Ref	–	Ref	–	Ref	–	Ref	–	Ref	–	Ref	–	Ref	–
	White other	1.15 (0.74 to 1.81)	0.53	1.71 (1.06 to 2.78)	0.03	1.09 (0.56 to 2.12)	0.80	1.43 (0.87 to 2.35)	0.15	1.04 (0.68 to 1.60)	0.85	1.44 (0.96 to 2.14)	0.08	0.94 (0.69 to 1.29)	0.70
	Black and minority ethnicity	0.82 (0.57 to 1.20)	0.31	1.60 (1.09 to 2.35)	0.02	1.34 (0.82 to 2.21)	0.24	1.18 (0.80 to 1.73)	0.40	0.82 (0.61 to 1.12)	0.22	0.97 (0.73 to 1.31)	0.86	0.82 (0.67 to 1.00)	0.05
	Overall	χ^2^(2) = 1.9	0.39	χ^2^(2) = 8.0	0.02	χ^2^(2) = 1.4	0.50	χ^2^(2) = 2.2	0.33	χ^2^(2) = 1.8	0.41	χ^2^(2) = 3.5	0.18	χ^2^(2) = 4.0	0.13
First language	Not English	Ref	–	Ref	–	Ref	–	Ref	–	Ref	–	Ref	–	Ref	–
	English	1.23 (0.79 to 1.91)	0.36	1.23 (0.77 to 1.96)	0.38	0.76 (0.43 to 1.33)	0.33	1.18 (0.74 to 1.89)	0.48	1.09 (0.75 to 1.60)	0.66	2.15 (1.44 to 3.19)	< 0.001*	1.16 (0.90 to 1.49)	0.26
Living alone	Not living alone	Ref	–	Ref	–	Ref	–	Ref	–	Ref	–	Ref	–	Ref	–
	Living alone	1.16 (0.86 to 1.57)	0.34	1.59 (1.10 to 2.30)	0.01	1.96 (1.27 to 3.01)	0.002*	1.61 (1.11 to 2.33)	0.01	1.14 (0.86 to 1.50)	0.36	1.23 (0.96 to 1.57)	0.10	1.17 (0.97 to 1.41)	0.10
Relationship status	Not partnered	Ref	–	Ref	–	Ref	–	Ref	–	Ref	–	Ref	–	Ref	–
	Partnered	0.83 (0.65 to 1.05)	0.12	0.82 (0.60 to 1.12)	0.21	0.49 (0.33 to 0.72)	< 0.001*	0.87 (0.64 to 1.17)	0.36	1.12 (0.89 to 1.40)	0.33	1.14 (0.93 to 1.41)	0.21	1.08 (0.93 to 1.25)	0.33
Ever had COVID–19	Think not	Ref	–	Ref	–	Ref	–	Ref	–	Ref	–	Ref	–	Ref	–
	Think yes	1.08 (0.84 to 1.40)	0.55	0.95 (0.68 to 1.33)	0.75	1.50 (1.05 to 2.16)	0.03	1.20 (0.90 to 1.60)	0.22	1.06 (0.84 to 1.32)	0.64	1.01 (0.82 to 1.23)	0.94	1.28 (1.14 to 1.44)	< 0.001*
Financial hardship	Range 3 (least) to 15 (most)	0.94 (0.91 to 0.98)	0.001*	1.01 (0.97 to 1.06)	0.54	0.98 (0.93 to 1.04)	0.52	0.99 (0.95 to 1.03)	0.66	0.95 (0.92 to 0.98)	0.001*	0.94 (0.91 to 0.96)	< 0.001*	0.94 (0.92 to 0.96)	< 0.001*

^†^Adjusting for wave, region, gender, age (raw and quadratic), presence of a dependent child in the household, having a chronic illness oneself, having a household member who has chronic illness, employment status, highest earner in household works in a manual occupation, index of multiple deprivation, highest educational or professional qualification, ethnicity, first language, living alone, relationship status, having had COVID-19 before, and financial hardship.

*p ≤ 0.004.

**Table 6:** Contextual and psychological factors associated with engaging in highest risk social mixing at different time points in the pandemic.

Attribute	Level	Rule of six indoors and outdoors		Second national lockdown		Third national lockdown		Rule of six outdoors, no indoor mixing		Rule of six indoors, up to 30 people outdoors		No legal restrictions		No legal obligation to self-isolate	
		aOR for engaging in highest risk social mixing (95% CI)^†^	*p*	aOR for engaging in highest risk social mixing (95% CI)^†^	*p*	aOR for engaging in highest risk social mixing (95% CI)^†^	*p*	aOR for engaging in highest risk social mixing (95% CI)^†^	*p*	aOR for engaging in highest risk social mixing (95% CI)^†^	*p*	aOR for engaging in highest risk social mixing (95% CI)^†^	*p*	aOR for engaging in highest risk social mixing (95% CI)^†^	*p*
Been out to work in last week	No	Ref	–	Ref	–	Ref	–	Ref	–	Ref	–	Ref	–	Ref	–
	Yes	1.34 (1.06 to 1.68)	0.01	1.14 (0.86 to 1.52)	0.37	1.76 (1.23 to 2.53)	0.002*	1.74 (1.30 to 2.34)	< 0.001*	1.23 (1.00 to 1.51)	0.06	1.20 (0.99 to 1.45)	0.06	1.99 (1.71 to 2.32)	< 0.001*
Worry about COVID–19	5-point scale (1 = not at all worried to 5 = extremely worried)	0.75 (0.69 to 0.82)	< 0.001*	0.65 (0.58 to 0.72)	< 0.001*	0.65 (0.57 to 0.75)	< 0.001*	0.67 (0.60 to 0.75)	< 0.001*	0.71 (0.65 to 0.77)	< 0.001*	0.76 (0.71 to 0.81)	< 0.001*	0.71 (0.67 to 0.75)	< 0.001*
Perceived risk of COVID–19 to self	5-point scale (1 = no risk at all to 5 = major risk)	0.78 (0.71 to 0.85)	< 0.001*	0.65 (0.58 to 0.73)	< 0.001*	0.74 (0.64 to 0.85)	< 0.001*	0.72 (0.64 to 0.81)	< 0.001*	0.68 (0.62 to 0.74)	< 0.001*	0.81 (0.75 to 0.87)	< 0.001*	0.76 (0.72 to 0.79)	< 0.001*
Perceived risk of COVID–19 to people in the UK	5-point scale (1 = no risk at all to 5 = major risk)	0.81 (0.74 to 0.89)	< 0.001*	0.62 (0.55 to 0.70)	< 0.001*	0.60 (0.52 to 0.70)	< 0.001*	0.71 (0.63 to 0.80)	< 0.001*	0.78 (0.71 to 0.85)	< 0.001*	0.82 (0.76 to 0.89)	< 0.001*	0.77 (0.73 to 0.82)	< 0.001*
Coronavirus would be a serious illness for me	5-point scale (1 = strongly disagree to 5 = strongly agree)	0.75 (0.69 to 0.82)	< 0.001*	0.61 (0.54 to 0.68)	< 0.001*	0.71 (0.61 to 0.82)	< 0.001*	0.65 (0.58 to 0.73)	< 0.001*	0.73 (0.67 to 0.79)	< 0.001*	0.79 (0.73 to 0.85)	< 0.001*	0.73 (0.69 to 0.77)	< 0.001*
I would worry about what others would think of me if I tested positive for coronavirus	5-point scale (1 = strongly disagree to 5 = strongly agree)	0.87 (0.81 to 0.95)	0.001*	0.85 (0.76 to 0.94)	0.002*	0.90 (0.79 to 1.03)	0.12	0.91 (0.82 to 1.00)	0.06	0.88 (0.82 to 0.95)	0.001*	0.81 (0.76 to 0.87)	< 0.001*	0.83 (0.79 to 0.87)	< 0.001*
Someone could spread coronavirus to other people, even if they do not have symptoms yet	5-point scale (1 = strongly disagree to 5 = strongly agree)	0.92 (0.82 to 1.03)	0.13	0.72 (0.63 to 0.82)	< 0.001*	0.68 (0.57 to 0.80)	< 0.001*	0.75 (0.66 to 0.86)	< 0.001*	0.86 (0.78 to 0.95)	0.004*	0.97 (0.89 to 1.06)	0.52	1.00 (0.94 to 1.07)	0.98
My personal behaviour has an impact on how coronavirus spreads	5-point scale (1 = strongly disagree to 5 = strongly agree)	0.83 (0.76 to 0.91)	< 0.001*	0.74 (0.67 to 0.82)	< 0.001*	0.74 (0.64 to 0.85)	< 0.001*	0.70 (0.63 to 0.78)	< 0.001*	0.88 (0.81 to 0.95)	0.001*	0.93 (0.87 to 1.00)	0.05	0.91 (0.86 to 0.96)	< 0.001*
I think the risks of coronavirus are being exaggerated	5-point scale (1 = strongly disagree to 5 = strongly agree)	1.19 (1.10 to 1.28)	< 0.001*	1.47 (1.33 to 1.62)	< 0.001*	1.42 (1.25 to 1.61)	< 0.001*	1.37 (1.24 to 1.51)	< 0.001*	1.18 (1.09 to 1.27)	< 0.001*	1.14 (1.06 to 1.22)	< 0.001*	1.15 (1.10 to 1.21)	< 0.001*
Information from the UK Government about coronavirus can be trusted	5-point scale (1 = strongly disagree to 5 = strongly agree)	0.84 (0.77 to 0.90)	< 0.001*	0.79 (0.71 to 0.88)	< 0.001*	0.84 (0.74 to 0.96)	0.009	0.79 (0.71 to 0.87)	< 0.001*	0.85 (0.79 to 0.92)	< 0.001*	0.86 (0.80 to 0.91)	< 0.001*	0.92 (0.88 to 0.97)	0.001*
Information from the UK Government about coronavirus is biased or one-sided	5-point scale (1 = strongly disagree to 5 = strongly agree)	1.03 (0.95 to 1.12)	0.51	1.05 (0.94 to 1.18)	0.37	1.17 (1.02 to 1.35)	0.03	1.08 (0.97 to 1.20)	0.17	1.07 (0.99 to 1.15)	0.10	0.98 (0.92 to 1.05)	0.60	0.95 (0.90 to 1.00)	0.04

^†^Adjusting for wave, region, gender, age (raw and quadratic), presence of a dependent child in the household, having a chronic illness oneself, having a household member who has chronic illness, employment status, highest earner in household works in a manual occupation, index of multiple deprivation, highest educational or professional qualification, ethnicity, first language, living alone, relationship status, having had COVID-19 before, and financial hardship.

*p ≤ 0.004.

At most timepoints (five or six out of seven), engaging in highest risk social mixing was associated with younger age, not agreeing that you would worry about what others would think of you if you tested positive for COVID-19, not agreeing that your personal behaviour had an impact on how COVID-19 spread, and not agreeing that information from the UK Government about COVID-19 could be trusted (Tables 5, 6). Lower financial hardship and not agreeing that someone could spread COVID-19 even if asymptomatic were associated with engaging in highest risk social mixing at four timepoints, while having been out to work was associated with engaging in highest risk social mixing at three time points (Tables 5, 6).

Men were more likely to engage in highest risk social mixing during the third lockdown and after the legal obligation to self-isolate if symptomatic or positive for SARS-CoV-2 was removed (Table 5).

In the third national lockdown, engaging in highest risk social mixing was associated with living alone and not having a partner (Table 5). When the rule of six was in place outdoors but there was no indoor mixing, later survey wave was associated with engaging in highest risk social mixing (Table 5). When there were no legal restrictions on social mixing, speaking English as your first language was associated with engaging in highest risk social mixing (Table 5).

When there was no legal obligation to self-isolate if symptomatic or positive for SARS-CoV-2, highest risk social mixing was associated with thinking that you had already had COVID-19 and living in a more deprived area; having a chronic illness was associated with not engaging in highest risk social mixing (Tables 5, 6).

Correlations between psychological factor items are reported in Supplementary Materials 4. Most items were significantly correlated.

## Discussion

We computed a composite measure of social mixing associated with a higher risk of SARS-CoV-2 transmission, taking into account setting, physical distancing, number of households present, and number of people from other households present. We described how people’s social mixing patterns varied between September 2020 and April 2022. This measure of social mixing shows clear variation in line with UK Government guidance in place at the time of data collection, including low rates of any social mixing during the third national lockdown, an increase in lower risk socialising (outdoors) between April and May 2021, and an increase in highest risk socialising (indoors) following the opening of indoor hospitality and removal of any legal restrictions on social mixing. Our analyses replicate findings investigating contact patterns during the pandemic^4^, and extend them by suggesting that changes occurred not only in the number, but also in the nature, of social interactions.

Psychological factors were consistently associated with engaging in highest risk social mixing. Similar to other studies, we found that highest risk social mixing was associated with lower perceived risk of COVID-19^20^, being less worried about COVID-19^21,22^, and lower perceived severity of COVID-19^4,11^. As in other pandemics, believing that the risks of the virus were being exaggerated was associated with not engaging with protective behaviours^23^. While strong and consistent associations between worry about, perceived risk and perceived severity of illness suggest that increasing worry and risk may result in increased protective behaviours, messaging designed to increase worry and perception of risk should only be contemplated when perceived risk is disproportionately low and in tandem with messaging highlighting the effectiveness of protective behaviours^24^.

Lower knowledge that SARS-CoV-2 can spread even when asymptomatic was consistently associated with engaging in highest risk social mixing. Other studies have also shown that people with lower knowledge of transmission are less likely to intend to adhere to the test, trace, and isolate system^25^. People who agreed that their personal behaviour had an impact on how COVID-19 spreads were less likely to engage in highest risk social mixing. This is in line with other evidence on internal health locus of control and uptake of health behaviours^14,26–28^. Increasing knowledge about SARS-CoV-2 transmission and emphasising that an individual’s behaviour can affect the spread of the virus may encourage uptake of protective behaviours such as lower risk social mixing. Associations between highest risk social mixing and disagreeing that information about COVID-19 from the UK Government can be trusted suggest that messaging about such issues may be better received if it is communicated by sources other than the Government, such as the National Health Service (NHS) or public health agencies.

Highest risk social mixing was associated with socio-demographic characteristics, namely younger age, having been out to work, and lower financial hardship. These findings may in part reflect the fact that younger people have higher rates of social contacts^29,30^, and that people who attend work have on average twice the number of social contacts than those working from home^31^. It also follows that, if people have been in close contact with others in the workplace, they may be more comfortable and perceive less risk in socialising outside of work. People in greater financial hardship may be less likely to be able to afford costs associated with frequent higher risk socialising (e.g. transport to and from meeting points, eating or drinking at hospitality venues).

After the removal of all legal restrictions on socialising on 19 July 2021, the UK Government moved to a system where individuals were expected to understand and manage their own risk, rather than follow rules^8^. However, risk perception is complex^32^, and evidence suggests that people have difficulty interpreting their own risk in different situations^33^. It is notable that when the legal obligation to self-isolate if symptomatic or positive for SARS-CoV-2 was removed, a time where SARS-CoV-2 cases in England were high, people with chronic health conditions were less likely to engage in highest risk social mixing, perhaps pointing to a continuing impact of high risk perception among this group. While messaging may encourage people to engage in less risky social mixing, our evidence and that from elsewhere suggests that the biggest driver of mixing behaviour is the restrictions in place at the time^34–37^. If they are needed again, obtaining large reductions in mixing as a result of communication alone may prove challenging.

Strengths of this study include that it gives a nuanced insight into social mixing behaviour over the course of the pandemic, spanning different restrictions, including the removal of all legal restrictions on social mixing. Limitations include: (1) the use of an online sample, whose views and behaviours may not be representative of the wider population, although associations within the data should remain valid^38^. (2) Surveys were cross-sectional, therefore we cannot imply causation. (3) We asked participants for details about the most recent time they met with people from another household to minimise recall bias. As surveys were conducted at the start of the week (usually Monday to Tuesday or Wednesday), people’s most recent instance of social mixing was likely to have been during the preceding weekend. Socialising patterns during the week may be different. (4) Participants were asked about their behaviour in the previous week. In some cases, this may have overlapped with a change in restrictions. (5) Patterns of social mixing may not be as significant predictors of COVID-19 risk as other aspects of a person’s life, such as their work environment, commuting and interactions with social and healthcare. (6) We did not include vaccination status as an explanatory variable in our regression analyses. All UK adults became eligible to have the first dose of the COVID-19 vaccine on 17 June 2021 (with their second dose 8 weeks after)^39^. Before this date, only certain age groups were eligible, which would have confounded analyses. To keep analyses across time points consistent, we did not include vaccination status in our final time point.

This study outlines patterns of social mixing between September 2020 and April 2022 using a composite measure drawing together information about factors influencing transmission risk (setting, distancing, and number of other households and individuals from other households present). Mixing behaviour varied according to the restrictions in place at the time. Messages targeting psychological factors, such as increasing knowledge about SARS-CoV-2 transmission and that an individual’s behaviour can impact the spread of the virus, may promote lower risk social mixing. However, should Government deem it necessary to significantly reduce risky social mixing in a short space of time, it is likely that a reimposition of restrictions may be necessary.

## Supplementary Information

Supplementary Information.

## Data Availability

The data are owned by the UK’s Department of Health and Social Care, so no additional data are available from the authors.
