# Supplementary material for: Patterns of social mixing in England changed in line with restrictions during the COVID-19 pandemic (September 2020 to April 2022)
Source: Sci Rep. 2022 Jun 21;12:10436. doi: 10.1038/s41598-022-14431-3 (PMC9212204; doi:10.1038/s41598-022-14431-3)
Supplement: Supplementary file 1 — Supplementary Information. [file 41598_2022_14431_MOESM1_ESM.docx]

**Supplementary materials 1. Detailed description of UK restrictions**

People asked to stay at home, 16 March 2020 ^1^

- Stay at home if you or anyone in your household has a high temperature or a new and continuous cough.
- Stop non-essential contact with others and all unnecessary travel.
- Start working from home if you can.
- Avoid pubs, clubs, theatres and other such social venues

First national lockdown, 23 March 2020 ^2^

- People only allowed to leave home for very limited purposes:
  - shopping for basic necessities, as infrequently as possible
  - one form of exercise a day;
  - any medical need, to provide care or to help a vulnerable person; and
  - travelling to and from work, only where it cannot be done from home.
- Shops selling non-essential goods and other premises including libraries, playgrounds and outdoor gyms, and places of worship closed.
- All public gatherings of more than two people stopped.
- All social events stopped, including weddings, baptisms, and other ceremonies.

Step 1 of lockdown restrictions easing, 13 May 2020 ^3^

- Can spend time outdoors and exercise as often as you like.
- Can meet one person outside of your household outside (outdoors), provided you stay 2 metres apart.

Step 2 of lockdown restrictions easing, 1 June 2020 ^4^

- Groups of up to six people able to meet outdoors, including in gardens and other private outdoor spaces, providing strict social distancing guidelines are followed.

Step 3 of lockdown restrictions easing, 4 July 2020 ^5^

- “One metre plus” where it is not possible to stay two metres apart (one metre apart, plus mitigations which reduce the spread of transmission e.g. installing screens, wearing a face covering).
- Premises allowed to reopen, including hotels, places of worship, libraries, hospitality venues, museums and galleries, hair salons and barbers, outdoor playgrounds and gyms.
- Household allowed to meet with one other household at a time, including staying over (can be indoors or outside, in a private home, restaurant or pub, or in paid accommodation).

Rule of six, 14 September 2020 ^6^

- Not allowed to meet with people from other households socially in groups of more than six (indoors or outdoors)

Tiers (local COVID-19 alert level), 14 October 2020 ^7,8^

- 1 (medium)
  - Not allowed to socialise in groups of more than six (“rule of six”)
  - Bars, pubs and clubs closed between 10pm and 5am
- 2 (high)
  - Not allowed to socialise with others outside their household in an indoor setting
  - Not allowed to socialise in groups of more than six in an outdoor setting, including gardens
  - Bars, pubs and clubs closed between 10pm and 5am
- 3 (very high)
  - Not allowed to socialise with others outside their household in an indoor setting
  - Not allowed to socialise in groups of more than six in an outdoor private setting, including gardens
  - Not allowed to socialise with others outside their household in an indoor setting
  - Can socialise in groups of more than six in an outdoor public setting, including parks
  - People inside these areas should avoid travelling outside these areas and staying overnight in other parts of the UK

Second national lockdown, 5 November 2020 ^9^

- People only allowed to leave home for very limited purposes:
  - for education;
  - for work, if you cannot work from home;
  - for exercise and recreation outdoors, with your household, or on your own with one person from another household;
  - for all medical reasons, appointments and to escape injury or harm;
  - to shop for food and essentials; and
  - to provide care for vulnerable people, or as a volunteer.
- Non-essential shops, leisure and entertainment venues closed.
- Pubs, bars, restaurants closed, except for takeaway and delivery services.
- People should work from home wherever possible.

Tiers (local COVID-19 alert level), 2 December 2020 ^10,11^

- 1 (medium)
  - Work from home where possible
  - Not allowed to socialise in groups of more than six (“rule of six”)
  - Collective worship, shops, personal care, gyms and leisure sector reopens.
- 2 (high)
  - Not allowed to socialise with others outside their household in an indoor setting
  - Not allowed to socialise in groups of more than six in an outdoor setting, including gardens
  - Alcohol only served in hospitality settings as part of a substantial meal.
- 3 (very high)
  - Not allowed to socialise with others outside their household in an indoor setting
  - Not allowed to socialise in groups of more than six in an outdoor private setting, including gardens
  - Not allowed to socialise with others outside their household in an indoor setting
  - Can socialise in groups of more than six in an outdoor public setting, including parks
  - People inside these areas should avoid travelling outside these areas and staying overnight in other parts of the UK
  - Indoor entertainment, hotels, and hospitality (except for delivery and takeaways) closed

Tier 4 introduced, 19 December 2020 ^12^

- “Stay at home”
- People not allowed to enter or leave Tier 4 areas, and Tier 4 residents not allowed to stay overnight away from home.
- Non-essential retail, indoor entertainment and personal care sectors closed.
- No social contact apart from meeting one other person in an outdoor public space.

Third national lockdown, 5 January 2021 ^13^

- People only allowed to leave their homes for the following reasons:
  - shop for basic necessities, for yourself or a vulnerable person
  - go to work, or provide voluntary or charitable services, if you cannot reasonably do so from home
  - exercise with your household or one other person, this should be limited to once per day, and you should not travel outside your local area
  - seek medical assistance or avoid injury, illness or risk of harm (including domestic abuse); and
  - attend education or childcare - for those eligible.
- All primary schools, secondary schools and colleges will move to remote learning, except for the children of key workers and vulnerable children.
- All non-essential retail, hospitality and personal care services closed.
- Restaurants closed, except for delivery, takeaway or click-and-collect of food and non-alcoholic drinks (but no longer for alcohol).
- Essential shops and garden centres remained open. Entertainment venues and playgrounds remained open.
- Places of worship remain open, but you could only visit with your household.
- Indoor and outdoor sports facilities closed but elite sport and disabled sport continued.

Step 1 of roadmap, 29 March 2021 ^14^

- Not allowed to socialise with others outside their household in an indoor setting.
- Not allowed to socialise in groups of more than six, or two households, in an outdoor setting.
- Outdoor sports facilities reopen.
- Overseas travel banned.

Step 2 of roadmap, 12 April 2021 ^14,15^

- Non-essential retail, personal care premises, and public buildings reopen.
- Most outdoor attractions reopen.
- Indoor leisure facilities reopen, but only for use by people on their own or with their household.
- Hospitality venues can serve people outdoors only.
- Self-contained accommodation, such as holiday lets, where indoor facilities are not shared with other households, reopen.

Step 3 of roadmap, 17 May 2021 ^14,16^

- Outdoors, most social contact rules lifted, but gatherings of over 30 people remained illegal.
- Not allowed to socialise with more than six people from outside your household in an indoor setting
- Indoor hospitality, entertainment venues, the rest of the accommodation sector, and indoor adult group sports and exercise classes reopen.
- Larger performances and sporting events in indoor venues with a capacity of 1,000 people or half-full (whichever is lower) allowed, as were those in outdoor venues with a capacity of 4000 people or half-full (whichever is lower).
- In the largest outdoor seated venues where crowds can spread out, up to 10,000 people will be able to attend (or a quarter-full, whichever is lower).
- International travel can begin.

Step 4 of roadmap, 19 July 2021 ^14,17^

- All legal limits on social contact removed.
- Wearing a face covering in certain indoor spaces no longer a legal obligation.

References

1 Prime Minister's Office. *Prime Minister's statement on coronavirus (COVID-19): 16 March 2020*, <https://www.gov.uk/government/speeches/pm-statement-on-coronavirus-16-march-2020> (2020).

2 Prime Minister's Office. *Prime Minister's statement on coronavirus (COVID-19): 23 March 2020*, <https://www.gov.uk/government/speeches/pm-address-to-the-nation-on-coronavirus-23-march-2020> (2020).

3 Prime Minister's Office. *Prime Minister's statement on coronavirus (COVID-19): 11 May 2020*, <https://www.gov.uk/government/speeches/pm-statement-on-coronavirus-11-may-2020#:~:text=You%20can%20now%20spend%20time,you%20stay%202%20metres%20apart> (2020).

4 Prime Minister's Office. PM: Six people can meet outside under new measures to ease lockdown, <https://www.gov.uk/government/news/pm-six-people-can-meet-outside-under-new-measures-to-ease-lockdown> (2020).

5 Prime Minister's Office. *Prime Minister's statement on coronavirus (COVID-19): 23 June 2020*, <https://www.gov.uk/government/speeches/prime-ministers-statement-on-coronavirus-covid-19-23-june-2020> (2020).

6 Cabinet Office. *Coronavirus (COVID-19): What has changed – 9 September*, <https://www.gov.uk/government/news/coronavirus-covid-19-what-has-changed-9-september> (2020).

7 Prime Minister's Office. *Prime Minister announces new local COVID Alert Levels*, <https://www.gov.uk/government/news/prime-minister-announces-new-local-covid-alert-levels> (2020).

8 House of Lords Library. *Covid-19 local alert levels: Three-tier system for England*, <https://lordslibrary.parliament.uk/covid-19-local-alert-levels-three-tier-system-for-england/> (2020).

9 Prime Minister's Office. *Prime Minister announces new national restrictions*, <https://www.gov.uk/government/news/prime-minister-announces-new-national-restrictions> (2020).

10 Prime Minister's Office. *PM statement on COVID-19 Winter Plan: 23 November 2020*, <https://www.gov.uk/government/speeches/pm-statement-on-covid-19-winter-plan-23-november-2020> (2020).

11 House of Lords Library. *Covid-19: Revised tiers for England*, <https://lordslibrary.parliament.uk/covid-19-revised-tiers-for-england/> (2020).

12 Prime Minister's Office. *Prime Minister announces Tier 4: 'Stay At Home' Alert Level in response to new COVID variant*, <https://www.gov.uk/government/news/prime-minister-announces-tier-4-stay-at-home-alert-level-in-response-to-new-covid-variant> (2020).

13 Prime Minister's Office. *Prime Minister announces national lockdown*, <https://www.gov.uk/government/news/prime-minister-announces-national-lockdown> (2021).

14 Cabinet Office. *COVID-19 Response - Spring 2021 (Summary)*, <https://www.gov.uk/government/publications/covid-19-response-spring-2021/covid-19-response-spring-2021-summary> (2021).

15 Prime Minister's Office. *Further easing of Covid restrictions confirmed for 12 April*, <https://www.gov.uk/government/news/further-easing-of-covid-restrictions-confirmed-for-12-april> (2021).

16 Prime Minister's Office. *Further easing of COVID restrictions confirmed for 17 May*, <https://www.gov.uk/government/news/further-easing-of-covid-restrictions-confirmed-for-17-may> (2021).

17 Prime Minister's Office. *Prime Minister confirms move to Step 4*, <https://www.gov.uk/government/news/prime-minister-confirms-move-to-step-4> (2021).

# Supplementary materials 2. Survey questions

SINGLE RESPONSE

**S1. Could you please provide your full UK postcode? Please ensure to include a space where applicable, e.g. AB1 2CD**

SINGLE CODE

**S2. What is your gender?**

- Male
- Female
- Prefer to self-describe
- Prefer not to say

NUMBER RESPONSE

**S3. Please can you tell me your age at your last birthday?**

_________________________

SCREENOUT IF AgeInt <16

SINGLE CODE

**Q1. Overall, how worried are you about coronavirus?**

- Extremely worried
- Very worried
- Somewhat worried
- Not very worried
- Not at all worried
- Don’t know

GRID QUESTION. SINGLE CODE PER STATEMENT

**Q2. To what extent do you think coronavirus poses a risk to:**

SCALE:

- Major risk
- Significant risk
- Moderate risk
- Minor risk
- No risk at all
- Don’t know

STATEMENTS:

- - People in the UK?
  - To you personally?

GRID QUESTION. SINGLE CODE PER STATEMENT

**Q3. To what extent do you agree or disagree with the following statements:**

SCALE:

- Strongly agree
- Agree
- Neither agree nor disagree
- Disagree
- Disagree strongly
- Don’t know

STATEMENTS:

- - Information from the Government about coronavirus can be trusted
  - Information from the Government about coronavirus is biased or one-sided

ASK ALL

GRID QUESTION. SINGLE CODE PER STATEMENT. RANDOMISE STATEMENTS.

**Q4. To what extent do you agree or disagree with the following statements:**

SCALE:

- Strongly agree
- Agree
- Neither agree nor disagree
- Disagree
- Strongly disagree
- Don’t know

STATEMENTS:

- Coronavirus would be a serious illness for me
- I would worry about what others would think of me if I tested positive for coronavirus
- Someone could spread coronavirus to other people, even if they do not have symptoms yet
- My personal behaviour has an impact on how coronavirus spreads
- I think the risks of coronavirus are being exaggerated

*Waves 28 to 50*

GRID QUESTION. SINGLE CODE PER STATEMENT

**Q5. Please enter the number of times you have been out of your home in the last seven days, for each of the following reasons?**

**If you have not left your home for this reason, please write 0**

RANDOMISE ORDER, ANCHOR OTHER

STATEMENTS

- To go out to work
- To meet up with friends and/or family that you don’t live with

OPEN - NUMERICAL VALUE ONLY

*Waves 51 to 72*

GRID QUESTION. SINGLE CODE PER STATEMENT

**Q5. How many times have you done each of the following activities in the past seven days?**

**Please give an approximate number if you are unsure. If you have not done the activity at all in the last seven days, please write 0.**

RANDOMISE ORDER STATEMENTS

- Left the house to go out to work (number of days)
- Met up with friends and/or family that you don’t live with

OPEN - NUMERICAL VALUE ONLY

ASK IF Q5 VALUE 1+ = To meet up with friends and/or family that you don’t live with. SINGLE CODE

**Q6. And still thinking about only the last occasion you met up with friends and/or family, were you indoors or outdoors?**

- Exclusively outdoors
- Mostly outdoors
- Equally split between indoors and outdoors
- Mostly indoors
- Exclusively indoors

ASK IF Q5 VALUE 1+ = To meet up with friends and/or family that you don’t live with. SINGLE CODE

**Q7. And still thinking about the last time you met friends and/or family that you don’t live with, how many people from outside your household were there?**

OPEN ENDED, allow number input

ASK IF Q5 VALUE 1+ = To meet up with friends and/or family that you don’t live with. SINGLE CODE

**Q8. The last time you met with friends and/ or family that you don’t live with, how many households (not people) did those people come from?**

*Don’t include your own household in this number.*

OPEN ENDED, allow number input

ASK IF Q5 VALUE 1+ = To meet up with friends and/or family that you don’t live with. SINGLE CODE

**Q9. Again, thinking about the last occasion you met with friends and/or family that you don’t live with, did people stay at least 2m apart?**

- Yes - at all times
- Yes - most of the time
- Yes – some of the time
- No – not at all

SINGLE CODE

**Q10. Do you know if you have ever had, or currently have, coronavirus?**

- I’ve definitely had it, and had it confirmed by a test
- I think I’ve probably had it
- I don’t know whether I’ve had it or not
- I think I’ve probably not had it
- I’ve definitely not had it

GRID QUESTION. SINGLE CODE PER STATEMENT. RANDOMISE STATEMENTS

**Q11. Thinking now about the past seven days, could you tell us to what extent you agree or disagree with the following statements?**

SCALE:

- Strongly agree
- Agree
- Neither agree nor disagree
- Disagree
- Strongly disagree
- Not applicable

STATEMENTS:

- - I am finding my current living situation difficult
  - I am struggling to make ends meet
  - I am skipping meals I would usually have

SINGLE CODE

**Q12. How many people currently live in your household? Please select one answer**

*Please include yourself and all adults and children – including those not related to you*

- I live alone
- 2
- 3 – 4
- 5 – 6
- 7 +

MULTI CODE

**Q13.** **What age are any dependent children in your household? Please select all that apply**

*Dependent children are those aged under 18 living in your household.*

| No dependent children in household | 1 |
| --- | --- |
| 0 – 4 years old | 2 |
| 5 – 10 years old | 3 |
| 11 – 15 years old | 4 |
| 16 – 18 years old | 5 |

SINGLE CODE

**Q14. What is your marital status?**

- Single, never married
- Married
- Separated
- Divorced
- Widowed
- Partnered/in a relationship
- Prefer not to say

SINGLE CODE

**Q15**. **What is your employment status**?

- Full time paid job (31+ hours)
- Part time paid job (<31 hours)
- Doing paid work on a self-employed basis or within your own business
- Student / On a government training programme (Nation Traineeship/Modern Apprenticeship)
- Out of work (6 months or less)
- Out of work (more than 6 months)
- Looking after home / Homemaker
- Retired
- Disabled OR Long-term sick
- Unpaid work for a business, community or voluntary organisation
- Prefer not to say

SINGLE CODE

**Q16.** **Which of the following best describes the occupation of the member of your household with the largest income (the chief income earner)? Please select one answer**

Please indicate to which occupational group the Chief Income Earner in your household belongs, or which group fits best.

The Chief Income Earner is the person in your household with the largest income.

If the Chief Income Earner is retired and has an occupational pension please answer for their most recent occupation.

If the Chief Income Earner is not in paid employment but has been out of work for less than 6 months, please answer for their most recent occupation

- Semi or unskilled manual work (e.g. Manual workers, all apprentices to skilled trades, Caretaker, Park keeper, non-HGV driver, shop assistant)
- Skilled manual worker (e.g. Skilled Bricklayer, Carpenter, Plumber, Painter, Bus/ Ambulance Driver, HGV driver, AA patrolman, pub/bar worker, etc.)
- Supervisory or clerical/ junior managerial/ professional/administrative (e.g. Office worker, Student Doctor, Foreman with 25+ employees, salesperson, etc.)
- Intermediate managerial/ professional/ administrative (e.g. Newly qualified (under 3 years) doctor, Solicitor, Board director small organisation, middle manager in large organisation, principle officer in civil service/local government)
- Higher managerial/ professional/ administrative (e.g. Established doctor, Solicitor, Board Director in a large organisation (200+ employees, top level civil servant/public service employee)
- Student
- Casual worker – not in permanent employment
- Housewife/ Homemaker
- Retired and living on state pension
- Unemployed or not working due to long-term sickness
- Full-time carer of another household member
- Other

MULTI CODE

**Q17. Do you, or anyone else in your household have any long-standing illness, disability or infirmity?**

*Please select all that apply*

| 1 | Yes, I do |
| --- | --- |
| 2 | Yes, another household member |
| 3 | No [EXCLUSIVE] |
| 97 | Prefer not to say [EXCLUSIVE] |

SINGLE CODE

**Q18. Which of the following categories would best describe your ethnicity?**

White

- British/English/Welsh/Scottish/Northern Irish
- Irish
- Gypsy, Traveller or Irish Traveller
- Any other White background

Mixed/ Multiple ethnic groups

- White and Black Caribbean
- White and Black African
- White and Asian
- Any other Mixed/ Multiple ethnic background

Asian or Asian British

- Indian
- Pakistani
- Bangladeshi
- Chinese
- Any other Asian background

Black or Black British

- African
- Caribbean
- Any other Black/ African/ Caribbean background

Other ethnic group

- Arab
- Other
- Don’t know
- Prefer not to say

SINGLE RESPONSE

**Q19. What is your first language?**

**Please type in and select from the options below.**

[List of 68 options, plus “other – write in”]

SINGLE CODE

**Q20. What is the highest level of educational qualification you have received?**

- PHD/Doctor
- Masters
- Bachelor’s Degree or equivalent (Such as a NVQ level 5)
- Higher education (Such as a HND or a NVQ level 4)
- A level or equivalent (Such as Scottish Highers or NVQ level 3)
- GCSE and below (Such as O level or an RSA Diploma)
- Other qualifications (Such as NVQ level 1)
- No qualifications

# Supplementary materials 3. Histograms for variables included in composite measure.

Figure 1. Frequency of number of times respondents reported meeting up with friends and/or family that they did not live with in the last seven days.

Figure 2. Settings of respondents’ most recent social meeting.

Figure 3. Frequency of maintaining 2 metre distance from people from another household during respondents’ most recent social meeting.

Figure 4. Number of households present at respondents’ most recent social mixing (including own).

Figure 5. Number of people from other households present at respondents’ most recent social mixing.

# Supplementary materials 4. Correlations between psychological factor items

Table 1. Correlations between psychological factors in participants included at all time points.

|  |  | Worry about COVID-19 | Perceived risk of COVID-19 to self | Perceived risk of COVID-19 to people in the UK | Coronavirus would be a serious illness for me | I would worry about what others would think of me if I tested positive for coronavirus | Someone could spread coronavirus to other people, even if they do not have symptoms yet | My personal behaviour has an impact on how coronavirus spreads | I think the risks of coronavirus are being exaggerated | Information from the UK Government about coronavirus can be trusted | Information from the UK Government about coronavirus is biased or one-sided |
| --- | --- | --- | --- | --- | --- | --- | --- | --- | --- | --- | --- |
| Worry about COVID-19 | *r* | 1 | 0.64 | 0.62 | 0.50 | 0.24 | 0.24 | 0.28 | -0.31 | 0.14 | 0.002 |
|  | *p* | - | <.001* | <.001* | <.001* | <.001* | <.001* | <.001* | <.001* | <.001* | 0.69 |
|  | *n* | 29,243 | 28,934 | 28,915 | 28,515 | 29,029 | 28,947 | 29,005 | 28,964 | 28,779 | 28,493 |
| Perceived risk of COVID-19 to self | *r* |  | 1 | 0.63 | 0.58 | 0.16 | 0.22 | 0.25 | -0.29 | 0.13 | -0.002 |
|  | *p* |  | - | <.001* | <.001* | <.001* | <.001* | <.001* | <.001* | <.001* | 0.73 |
|  | *n* |  | 28,977 | 28,788 | 28,318 | 28,780 | 28,711 | 28,774 | 28,716 | 28,571 | 28,296 |
| Perceived risk of COVID-19 to people in the UK | *r* |  |  | 1 | 0.42 | 0.17 | 0.26 | 0.26 | -0.32 | 0.13 | -0.02 |
|  | *p* |  |  | - | <.001* | <.001* | <.001* | <.001* | <.001* | <.001* | 0.005 |
|  | *n* |  |  | 28,957 | 28,281 | 28,757 | 28,693 | 28,753 | 28,704 | 28,555 | 28,278 |
| Coronavirus would be a serious illness for me | *r* |  |  |  | 1 | 0.17 | 0.25 | 0.28 | -0.32 | 0.17 | -0.06 |
|  | *p* |  |  |  | - | <.001* | <.001* | <.001* | <.001* | <.001* | <.001* |
|  | *n* |  |  |  | 28,577 | 28,418 | 28,337 | 28,400 | 28,374 | 28,160 | 27,919 |
| I would worry about what others would think of me if I tested positive for coronavirus | *r* |  |  |  |  | 1 | -0.09 | 0.01 | 0.17 | 0.1 | 0.12 |
|  | *p* |  |  |  |  | - | <.001* | 0.08 | <.001* | <.001* | <.001* |
|  | *n* |  |  |  |  | 29,091 | 28,850 | 28,903 | 28,858 | 28,632 | 28,359 |
| Someone could spread coronavirus to other people, even if they do not have symptoms yet | *r* |  |  |  |  |  | 1 | 0.42 | -0.38 | 0.15 | -0.11 |
|  | *p* |  |  |  |  |  | - | <.001* | <.001* | <.001* | <.001* |
|  | *n* |  |  |  |  |  | 29,014 | 28,832 | 28,785 | 28,562 | 28,294 |
| My personal behaviour has an impact on how coronavirus spreads | *r* |  |  |  |  |  |  | 1 | -0.32 | 0.19 | -0.11 |
|  | *p* |  |  |  |  |  |  | - | <.001* | <.001* | <.001* |
|  | *n* |  |  |  |  |  |  | 29,073 | 28,838 | 28,623 | 28,351 |
| I think the risks of coronavirus are being exaggerated | *r* |  |  |  |  |  |  |  | 1 | -0.18 | 0.33 |
|  | *p* |  |  |  |  |  |  |  | - | <.001* | <.001* |
|  | *n* |  |  |  |  |  |  |  | 29,027 | 28,585 | 28,318 |
| Information from the UK Government about coronavirus can be trusted | *r* |  |  |  |  |  |  |  |  | 1 | -0.39 |
|  | *p* |  |  |  |  |  |  |  |  | - | <.001* |
|  | *n* |  |  |  |  |  |  |  |  | 28,827 | 28,323 |
| Information from the UK Government about coronavirus is biased or one-sided | *r* |  |  |  |  |  |  |  |  |  | 1 |
|  | *p* |  |  |  |  |  |  |  |  |  | - |
|  | *n* |  |  |  |  |  |  |  |  |  | 28,544 |

* *p*≤.004
